# Medical Physics Residency Consortium: collaborative endeavors to meet the ABR 2014 certification requirements

**DOI:** 10.1120/jacmp.v15i2.4490

**Published:** 2014-03-06

**Authors:** Brent C. Parker, John Duhon, Claus C. Yang, H. Terry Wu, Kenneth R. Hogstrom, John P. Gibbons

**Affiliations:** ^1^ Mary Bird Perkins Cancer Center Baton Rouge LA; ^2^ Department of Physics and Astronomy Louisiana State University and Agricultural & Mechanical College Baton Rouge LA; ^3^ e+ Oncologics, Inc. Lafayette LA; ^4^ University of Mississippi Medical Center Jackson MS; ^5^ Willis‐Knighton Cancer Center Shreveport LA USA

**Keywords:** residency training, board certification, CAMPEP accreditation

## Abstract

In 2009, Mary Bird Perkins Cancer Center (MBPCC) established a Radiation Oncology Physics Residency Program to provide opportunities for medical physics residency training to MS and PhD graduates of the CAMPEP‐accredited Louisiana State University (LSU)‐MBPCC Medical Physics Graduate Program. The LSU‐MBPCC Program graduates approximately six students yearly, which equates to a need for up to twelve residency positions in a two‐year program. To address this need for residency positions, MBPCC has expanded its Program by developing a Consortium consisting of partnerships with medical physics groups located at other nearby clinical institutions. The consortium model offers the residents exposure to a broader range of procedures, technology, and faculty than available at the individual institutions. The Consortium institutions have shown a great deal of support from their medical physics groups and administrations in developing these partnerships. Details of these partnerships are specified within affiliation agreements between MBPCC and each participating institution. All partner sites began resident training in 2011. The Consortium is a network of for‐profit, nonprofit, academic, community, and private entities. We feel that these types of collaborative endeavors will be required nationally to reach the number of residency positions needed to meet the 2014 ABR certification requirements and to maintain graduate medical physics training programs.

PACS numbers: 01.40.Fk, 01.40.gb

## I. INTRODUCTION

In October 2007, the American Board of Radiology (ABR) Board of Trustees approved a policy that will require completion of a CAMPEP‐approved residency program for candidates to be eligible for board certification. Known as the “2014 initiative”, the policy states: “Candidates taking Part 1 for the first time in 2014 or later also must have completed a CAMPEP‐accredited residency program before being eligible to take the Part 2 examination in Medical Physics.”^(1)^

In January 2009, the American Association of Physicists in Medicine (AAPM) sponsored a study, to be conducted by the Center for Health Workforce Studies at the School of Public Health at the State University of New York (SUNY) — Albany, of the medical physics workforce to better understand current supply and demand for the profession. The SUNY report calculated that the number of new radiation oncology medical physicists required to meet demand is projected to increase from 158 in 2010 to more than 190 by 2030.^(2)^ The authors reported a shortage of 20‐140 new medical physicists annually, depending on hw many CAMPEP‐accredited resident positions become available. The results of the SUNY report are consistent with a study by Mills et al.^(3)^ that indicated a demand of 150‐175 new radiation oncology physicists per year by 2020. Mills and colleagues estimated that, at a minimum, 125 new radiation oncology physicists per year will be required by 2020 to maintain the health of the medical physics profession. (Note: Such a projection could change based on changes in technology and medical practice; for example, Mills and colleagues thought it possible for time‐consuming procedures to become more efficient, in which case medical physics demand might be less by 2020.) Combining the results of the SUNY and the Mills workforce estimates, we assume that the profession will need to produce approximately 180 new radiation oncology physicists per year for the period 2020‐2030.

As of February 2013, there were 60 CAMPEP‐accredited radiation oncology physics residency programs with a total of approximately 84 new annual residency positions (approximately 1.4 new residency positions per program) (B. Gerbi, chair, CAMPEP Residency Education Program Review Committee, personal correspondence, February 2013). Assuming a need of 180 new radiation oncology physicists per year, there is currently a shortage of approximately 96 new annual positions. At 1.4 new annual positions per residency program, this equates to a need for 68 new residency programs.

**Figure 1:**
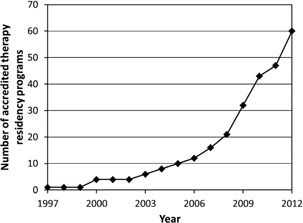
Number of accredited residency programs through 2012.

Figure 1 data from the CAMPEP Residency Education Program Review Committee show the growth in the number of accredited therapy residency programs from 1997‐2012. These data are consistent with that reported by Bayouth et al.,^(4)^ that from July 1, 2001 through July 2010, the number of accredited residency programs grew with a doubling time of 2.67 years. From 2009‐2012, a linear growth rate of ten programs per year is supported by the data. Assuming this linear rate remains constant, it will take approximately seven years to reach the goal of 180 new residency positions per year, well beyond the current 2014 ABR time frame.

There are also potential ethical issues with admitting students to graduate programs with no reasonable expectation that they will be able to find residency positions after graduation. This issue could impact graduate program recruiting and threaten the existence of some graduate programs in that students may elect to focus only on programs that offer real opportunities for residency positions. Hence, we have anticipated that some graduate programs will begin to align with residency programs.

Therefore, MBPCC made the strategic decision to establish a Radiation Oncology Physics Residency Program that could provide opportunities for resident positions to the graduates of the CAMPEP‐accredited LSU‐MBPCC graduate program in medical physics. This strategy was meant to protect its program from decline and possible demise, while helping ensure that it maintains the highest quality of matriculating students. However, since the American Association of Physicists in Medicine (AAPM) Report 90 recommends a physicist‐to‐resident ratio of 2:1,^(5)^ MBPCC (12 staff medical physicists) would only be able to accommodate six total residents (three new residents per year), or half of the average graduating class. It was therefore decided to establish a Consortium including a few select, nearby medical physics groups in order to accommodate three additional residents per year.

## II. MATERIALS AND METHODS

### A. Consortium structure

MBPCC (Baton Rouge, LA) established a Residency Consortium with medical physics groups at external regional institutions to provide clinical residency training. The Consortium structure is similar to the dependent affiliate model described in AAPM Report 133.^(6)^ Unlike the dependent affiliate model, however, each member of the Consortium provides a full range of clinical training as outlined in AAPM Report 90. Within the Consortium, MBPCC is responsible for program administration. These tasks include, but are not limited to, program governance and funding (internal and external), initial program accreditation, curriculum development, resident performance tracking, exam scheduling, and evaluation of residents. The partner sites are then able to focus on clinical training. The consortium model takes advantage of institutions with good clinical physics resources, but inadequate administrative resources to initiate and operate a large residency program of comparable quality.

To establish the Consortium, MBPCC first initiated its Residency Program in 2009, then it began approaching potential partners in early 2010. There was a high level of interest and resulting action from both the medical physicists and administrators at most of the targeted institutions to participate in radiation oncology physics resident training. The Consortium was established in late 2010 with a total of four institutions, MBPCC and three partner institutions. These partner institutions currently include e+ OncoLogics, Inc. (Lafayette, LA), Willis‐Knighton Cancer Center (Shreveport, LA), and the University of Mississippi Medical Center (Jackson, MS). The current Consortium membership includes private, community, for‐profit, nonprofit, and academic institutions. All current partner sites are within a four‐hour drive (250 miles) of MBPCC.

Institutions for each of the affiliate programs have an agreement with MBPCC. The agreement covers several areas, including MBPCC and affiliate responsibilities, resident program governance and marketing, resident placement, funding and insurance coverage, indemnification, and amendments or termination of agreement. Each agreement is signed by the MBPCC chief executive officer and an authorized representative from the affiliate site. A copy of a boiler‐plate agreement is available from MBPCC upon request.

### B. Consortium governance

The Consortium is governed by the Radiation Oncology Physics Residency Training Program Committee. The Program Committee is comprised of the Program Director, Deputy Program Director, the LSU Medical Physics Graduate Program Director (who is also the MBPCC Chief of Physics), an ABR‐certified radiation oncologist, a Medical Dosimetrist Certification Board (MDCB)‐certified medical dosimetrist, and an American Registry of Radiologic Technologists (ARRT)‐certified radiation therapist from MBPCC, as well as the affiliate Program Directors from each partner institution.

The Program Committee meets approximately once a month and provides input on a wide variety of issues regarding program operation. The affiliate Program Directors join the meeting via Skype (Skype Technologies S.A., Luxembourg) video conferencing. At each meeting, the Committee initially meets with the program's senior resident to discuss any concerns among the program residents. Afterwards, the resident is excused, and the Program Committee discusses other issues in the program. These include resident admissions and placement, resident progress and evaluations, program curriculum, budget (internal and external funding opportunities), accreditation, and promotion (e.g., website, public relations articles).

Additionally, each partner location has a local residency program committee that is responsible for training issues at the partner site. These local partner committees are chaired by the affiliate Program Director and are responsible for organizing the training schedules for the residents at each site. This also includes ensuring that resident progress is maintained in accordance to the Consortium training plan and curriculum.

### C. Resident recruitment and matching process

The program is designed to accept MS and PhD graduates of the Commission on Accreditation of Medical Physics Education Programs (CAMPEP)‐accredited, LSU‐MBPCC Medical Physics Program and post‐doctoral fellows who have completed a two‐to‐three‐year fellowship in radiation oncology physics and appropriate medical physics classes.^(7)^ LSU medical physics MS and PhD graduates and LSU and MBPCC post‐doctoral fellows have first priority for admission.

Initially, first priority applicants are matched to a Consortium training site based on the same algorithm used by the National Residency Matching Program (NRMP) for physician residents.^(8)^ Applicants interview at all Consortium sites to which they are willing to be matched. Once interviews are complete, each applicant ranks the sites at which he/she interviewed in order of preference, and each Consortium site ranks the applicants they interviewed in order of preference. To be matched to a site, an applicant must be ranked as “acceptable” by that site. This prevents a site from being forced to accept a candidate that they do not feel meets their minimum qualifications for residency training.

The NRMP algorithm is then applied to the rank lists to produce the best match of residents to training sites. Like the NRMP model, residents are not eligible to be matched to sites where they did not interview. Use of the NRMP algorithm ensures that the match process is unbiased and fair to all Consortium training sites. Results of the internal match are communicated to the applicants in mid‐January for positions starting on July 1. The matched applicants are then given 48 hours to accept, reject, or defer the offered position. Deferment indicates the candidate wishes to be reconsidered for this position (without priority) on the national offer date, if the position remains unfilled. Unfilled positions remaining after the acceptance period are then offered to the remaining internal candidates who have not been offered any positions, who are given 48 hours to decide. This process continues until all positions are filled or all internal candidates have reached a decision.

If any unfilled positions remain upon completion of the above process, they are opened to outside applicants. Outside applicants are interviewed and ranked along with internal candidates who elected to defer. These offers are extended on the national offer date in concordance with the current AAPM Working Group of Coordination of Medical Physics Residency Program's gentleman's agreement.

Once the Consortium reaches full operating capacity of six new residents per year, it will be large enough to accommodate all MS and PhD graduates of the LSU‐MBPCC Medical Physics Program. While graduates of the LSU‐MBPCC Medical Physics Program receive top priority in filling open residency positions, they are only guaranteed the opportunity for a residency position. The students must still demonstrate adequate performance in the graduate program, complete all degree requirements prior to beginning the residency program, and be acceptable to the offering institutions.

### D. Resident funding

Each resident is located at his matched training site for the duration of the two‐year residency. During this time, the Consortium sites fund their residents as employees of the institution.

Residents are currently funded at the same level as Post Graduate Years 1 and 2 (PGY‐1 and PGY‐2) in regional LSU system medical residencies, as originally recommended by AAPM Report 36.^(9)^ This level is also supported by a national survey in TG‐133, which indicated 73% of respondents favored funding at the PGY‐1 level.^(6)^ Each resident is also provided benefits including medical, dental, life insurance, 403(b), and paid time off. This model was chosen to maintain professional standing with our physician colleagues. Based on this salary structure, MBPCC can fund three to four residents for the cost of one board‐certified clinical medical physicist.

We estimate that the average resident clinical support contribution is 0.375 full‐time equivalent (FTE) clinical physicists. This was determined assuming 0.25 FTE for a first‐year resident and 0.5 FTE for a second‐year resident. This is slightly higher than the 0.25 FTe use in the study by Mills et al.^(3)^ Based on this estimate, the institutional cost per FTE clinical physics support for a physics resident is approximately the same as that for a staff clinical physicist.

Residents are credentialed at their training institutions after completing their first year of training. At this time, they are allowed to perform duties of a non‐ABR‐certified clinical physicist. Prior to credentialing, each resident must demonstrate competency for each task he/she will be performing independently. This competency evaluation is performed by the program faculty members who have been involved in the resident's training and must be approved by the chief clinical physicist at the site where the resident is located. Competency is demonstrated by successful completion of at least two one‐month rotations and passing an oral exam on the subject matter. Credentialing of second‐year residents serves two purposes. First, resident training is more cost‐effective, as the resident can contribute 0.5 FTE to clinical physics support; second, this allows the resident to become more comfortable with performing independent work. We feel that this provides an environment that more closely resembles the work environment in which the resident will be expected to function after entering the workforce.

In addition to institution funding, external funding has been received or is being sought. MBPCC was a recipient of the 2009 ASTRO/AAPM Radiation Oncology Physics Residency Training Program Grant. This provided seed funding for establishment of the residency program at MBPCC. The Consortium has also received funding from the Patrick Taylor Foundation,^(10)^ which has agreed to provide half‐support for two years for two residents based in Louisiana.

### E. Resident tracking and evaluation

As part of the Consortium, residents follow the same training plan regardless of training location. To ensure consistency in training across institutions, the Consortium has implemented a commercial software product (Typhon Group, LLC, Metairie, LA) to monitor resident performance and progress. The Typhon Group Allied Health Student Tracking module and its uses for medical physics residency program performance tracking have been previously described in detail by Zacarias and Mills.^(11)^

Tracking of resident time, tasks, clinical experiences, completed competencies, reports, and most evaluations is performed using the software from Typhon Group. The Program Director or his designee is responsible for managing the software, including updating the curriculum as needed. Residents are able to access the system and enter information on their program training requirements (e.g., completion of assigned competencies, attendance of required meetings, attended lectures). Program faculty have access to these data, as well as many reporting tools to analyze and approve entries.

Each resident typically meets daily with the staff physicist with whom they are rotating to discuss clinical duties for that day. Additionally, each resident is evaluated monthly by the program faculty member mentoring the resident on the project assignment. The project or rotation mentor meets with the resident each month to rview the topics covered during that month and to determine if any remediation is required. This prevents the resident from falling behind in his/her training. An evaluation survey has been created within the Typhon Group software to allow faculty members to evaluate resident performance for each clinical rotation or project that the faculty member supervises. The results of these monthly surveys are maintained within the Typhon software as part of the resident's permanent record.

Additionally, an evaluation survey has been created for residents to evaluate the assigned faculty member for each month of clinical rotation and for each assigned project. These surveys allow the Program Director to assess the need to assist faculty members in improving their resident training performance. Only the Program Director has access to the results of these surveys. Residents' names are not disclosed when the results of these surveys are communicated to faculty members. The results are also retained within the Typhon Group software as part of ongoing quality assurance of faculty performance and program training.

In addition to regular review by the faculty mentors, residents are evaluated by oral examination. The residents are examined by representatives from the host site and from two or more other Consortium sites via Skype video conferencing. The examinations ensure that residents are being held to the same training standards, regardless of location within the Consortium. Additionally, the oral examinations identify any deficiencies in resident knowledge so they may be addressed immediately. Currently, each resident is examined every four months, covering each of the independent projects and clinical rotations.

## III. RESULTS/DISCUSSION

### A. Program growth

The program growth was designed to reach the maximum of 12 total residents by 2016. This decision was made based on the fact that students entering the LSU graduate program in Fall 2012 would be able to enter the ABR certification pathway prior to the 2014 ABR mandate for completion of a residency program. Students entering the graduate program in Fall 2013 and completing degree requirement in Spring 2016 (LSU has an approximate three‐year, thesis‐based MS program) would be required to complete a residency program. Thus, by 2016 the Consortium would have to be able to accommodate all students completing the graduate program.

**Figure 2:**
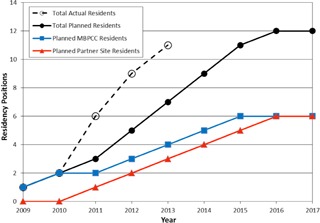
Planned and actual growth of number of residency positions.

In reality, the Consortium has grown faster than expected due to strong support by administrations and medical physics departments of the partner sites. All current partner sites began resident training in 2011. Predicted program growth indicates that the Consortium will reach full program capacity by July 2014, two years ahead of schedule. Figure 2 shows the planned and actual growth of the residency Consortium.

### B. CAMPEP accreditation

The residency program self‐study from the MBPCC Program was initially submitted to CAMPEP in October 2011. After discussions with CAMPEP, it was decided to submit the entire Consortium Program for accreditation at one time, rather than each affiliate site individually. The initial CAMPEP review of the self‐study primarily asked for additional documentation from the affiliate sites. A response was sent in February 2012, which included additional affiliate site materials and signed affiliate agreements. The CAMPEP site visit took place in June 2012.

This was the first residency model of this type to be reviewed by CAMPEP, and there was considerable discussion as to how best to site‐visit the program. As such, it was decided to have the three members of the site‐visit team spend one day at MBPCC, then split up the following day to visit a different affiliate site. The team then reconvened at the end of the second day in Baton Rouge and wrote the first draft of the report. The team reported its findings on the morning of the third day, before departing.

The program received full accreditation (through December 31, 2016) in August 2012. MBPCC and its affiliate sites each received individual certificates of accreditation and are listed on the CAMPEP website.

## IV. CONCLUSIONS

A consortium of regional medical physics groups has been formed to address a national short‐age of radiation oncology physics residency positions. This Consortium consists of Mary Bird Perkins Cancer Center, e+ OncoLogics, Inc., Willis‐Knighton Cancer Center, and the University of Mississippi Medical Center. The Consortium institutions have shown a great deal of support, both from their medical physics groups and administrations, in developing these partnerships. The details of these partnerships were finalized through affiliate agreements, signed by representatives from MBPCC and each affiliate institution. The Consortium is a network of for‐profit, nonprofit, academic, community, and private entities. We feel that these types of collaborative endeavors provide excellent residency training, and will be required to reach the number of residency positions needed to meet the 2014 ABR mandate and to maintain graduate medical physics training programs.

## ACKNOWLEDGMENTS

The authors acknowledge the contributions from other current and former members of the Program Committee, including Frank Apollo, CMD, Yolanda Augustus, RTT, Joseph Dugas, PhD, Kara Ferachi, MS, Wayne Newhauser, PhD, and Mary Ella Sanders, MD. The Consortium would not be possible without the ongoing enthusiastic contribution of professional staff (medical physicists, administrators, radiation oncologists, medical dosimetrists, and radiation therapists) at MBPCC and affiliate sites.
